# Association between serum minerals and malnutrition assessed by GLIM among hospitalized adults: a multicenter cross-sectional study

**DOI:** 10.3389/fnut.2026.1868158

**Published:** 2026-07-02

**Authors:** Hongmei Xue, Xinyu He, Huanyu Hu, Ying Wang, Ying Xie, Dandan Wang, Zhicui Yao, Chunhua Song, Wei Chen, Zengning Li

**Affiliations:** 1 Department of Clinical Nutrition, The First Hospital of Hebei Medical University, Shijiazhuang, China; 2 Hebei Key Laboratory of Nutrition and Health, Shijiazhuang, China; 3 School of Public Health, Hebei Medical University, Shijiazhuang, China; 4 Department of Nutrition, Affiliated Hospital of Hebei Engineering University, Handan, China; 5 School of Nursing, Hebei University of Chinese Medicine, Shijiazhuang, China; 6 Department of Epidemiology and Statistics, College of Public Health, Zhengzhou University, Zhengzhou, China; 7 Department of Clinical Nutrition, Peking Union Medical College Hospital, Peking Union Medical College, Chinese Academy of Medical Sciences, Beijing, China

**Keywords:** mineral, global leadership initiative on malnutrition, malnutrition, hospitalized, adult

## Abstract

**Objectives:**

To evaluate the relationship between single- and multiple-mineral deficiencies and malnutrition, as defined by the Global Leadership Initiative on Malnutrition (GLIM) criteria among Chinese inpatients.

**Methods:**

A multicenter cross-sectional study of hospitalized patients in hospitals was conducted in China. Information on the inpatients’ social demographic characteristics, physical examination, and laboratory test results was collected. Malnutrition was defined on the GLIM criteria. Multivariable logistic regression and restricted cubic spline were used to examine the relationship between individual serum minerals and their counts and malnutrition.

**Results:**

A total of 3,433 hospitalized adults were included in the study. Compared to those with normal mineral levels, patients with serum mineral deficiencies had a 1.43- to 2.31-fold higher likelihood of prevalent GLIM-based malnutrition. After stratifying by age, significant associations were observed only among middle-aged and elderly inpatients. For individuals aged 45 and older, low mineral levels were associated with a higher likelihood of malnutrition compared to normal levels; the ORs (95% CIs) ranged from 1.56 (1.01, 2.39) to 2.96 (1.71, 5.70). Furthermore, inpatients with one, two, and three or more minerals below the normal level had a respective 1.33-, 2.15-, and 2.95-fold higher likelihood of prevalent malnutrition, respectively.

**Conclusion:**

Our study found that serum mineral deficiencies—particularly potassium, sodium, chloride, and phosphorus—are significantly associated with prevalent malnutrition, with this association more pronounced among middle-aged and elderly patients. Moreover, the cumulative burden of mineral deficiencies showed a graded association with the likelihood of prevalent malnutrition, though this association requires further validation in prospective studies.

## 1 Introduction

Malnutrition is a significant issue for hospitalized adults, leading to various adverse health outcomes, such as prolonged hospital stays, decreased quality of life, increased risk of readmission, and higher mortality rates, along with escalating healthcare costs (1–5). According to the Global Leadership Initiative on Malnutrition (GLIM) criteria (6), which uses a two-step approach to diagnose malnutrition, its prevalence can vary widely, ranging from 16 to 80% (7). In China, the prevalence of malnutrition among hospitalized adults is reported to be 24.6% (5). Thus, addressing malnutrition in hospitalized patients requires urgent prioritization and rigorous investigation.

Approximately 65% people worldwide do not consume enough dietary minerals (8). Moreover, roughly 11–60% of hospitalized patients have mineral deficiencies (9–12), which are influenced by nutritional intake and play critical roles in human health—including cardiovascular health (13), COPD (14), diabetes, and insulin resistance (15), etc.—by regulating various physiological functions (16). However, studies regarding the relationship between mineral deficiencies and malnutrition are limited and inconsistent. Dietary minerals have been found to correlate with malnutrition in cancer patients (17). Yilmaz et al. (11) also concluded that nutritional status was not significantly associated with multiple micronutrient deficiencies in an observational study of 156 malnourished older adults. Yu et al. (18) collected blood samples from 149 patients and found that only the concentration of Calcium (Ca) may be an independent factor for high malnutrition risk. Furthermore, Eisenhut (19) revealed that malnutrition causes a reduction in alveolar epithelial sodium (Na) and chloride (Cl) transport, which predisposes to death from lung injury. Moreover, the malnutrition assessments in the aforementioned studies are also inconsistent.

Considering this, we conducted a multi-center survey on Chinese inpatients aged 18 years or older with seven systemic diseases to provide reliable data on the relationship of individual and multiple serum mineral deficiencies, which includes potassium (K), Na, Ca, magnesium (Mg), Cl, and phosphorus (P), to malnutrition diagnosed by the GLIM criteria.

## 2 Materials and methods

### 2.1 Study sample

Our survey was conducted in 19 hospitals in Hebei Province between August 2020 and October 2023. Hospitalized patients aged 18 years or older who were newly admitted to the hospital within 24 to 48 h (not emergency admissions) due to seven systemic diseases, including digestive, respiratory, cardiovascular, endocrine, nervous, and urinary system diseases, as well as cancers, were recruited. Participants who were critically ill patients, had mental illness and memory impairment, were unable to answer questions correctly, lacked capacity, etc., were excluded. Information on social-demographic characteristics, disease characteristics, physical examination within 24–48 h of admission, laboratory examination, body composition measurement, nutritional risk, and nutritional assessment was collected. Based on a validated case report form and workbook, all examinations and information extraction involved are performed by trained nutritionists or clinicians and then checked by professional team staff. The study protocol and procedures were approved by the Ethics Review Committee of Peking Union Medical College Hospital (No. ZS-2614). All the enrolled participants provided oral informed consent before the survey to confirm their voluntary participation.

Initially, 5,160 patients were recruited in this analysis. Of these, patients with missing plasma mineral data (*n* = 1707) and anthropometric indices (*n* = 20) were excluded from the present study. Baseline characteristics were then compared between included and excluded participants. Except for educational level (*p* < 0.0001), no statistically significant differences were found between groups regarding nutritional intervention, serum biochemical parameters, or socio-demographic indices (*p* ≥ 0.07) (Supplementary Table 1). Finally, a total of 3,433 patients were included, with 1918 males (55.87%) and 1,515 females (44.13%).

### 2.2 Nutritional risk and malnutrition assessment

The GLIM criteria are a two-step approach to diagnosing malnutrition (6). Firstly, “at risk” status was identified using any validated screening tool. Secondly, the diagnosis and severity of malnutrition were assessed. In the present study, the presence of nutritional risk was evaluated using the Nutritional Risk Screening 2002 (NRS 2002), as recommended by the European Society for Clinical Nutrition and Metabolism guidelines (20). It includes three components: the nutritional score (body mass index (BMI), weight loss, and dietary intake), the disease severity score, and the age score (age > 70 years). Patients are classified as having no or low risk when they have a total score < 3 or as having a moderate or high risk when they have a total score ≥ 3. Low BMI was adapted for the Asian population and was defined as a BMI < 18.5 kg/m^2^ for inpatients aged < 70 years and < 20.0 kg/m^2^ for inpatients aged ≥ 70 years (21).

For malnutrition assessment, at least one phenotypic indicator and one etiological indicator are required, based on the presence of nutritional risk (NRS 2002 ≥ 3 points) in the patients, according to GLIM criteria. Phenotypic criteria include non-volitional weight loss, low BMI, and reduced muscle mass, and two etiological criteria include reduced food intake, inflammation, or disease burden.

### 2.3 Minerals assessment

Serum minerals, including K, Na, Cl, Ca, Mg, and P (mmol/L for all), were measured by the Ion-Selective electrode method (K, Na, and Cl) and the colorimetric method (Ca, Mg, and P) using an Automated Clinical Chemistry Analyzer at each participating hospital. Although the specific analyzer models varied across the 19 hospitals, all laboratories adhered to the Chinese National Clinical Laboratory Standards for regular calibration and internal quality control. Blood samples were collected in the fasting state before intravenous fluid administration whenever clinically feasible, and all samples were processed within 2 h of collection to minimize pre-analytical variation. The reference ranges were harmonized across all 19 hospitals. We collected the actual numerical values reported by each hospital’s laboratory. Then we applied uniform cut-off points to define low, normal, and high mineral concentrations for the entire study population (22, 23): K: 3.6–5.2 mmol/L; Na: 136–146 mmol/L; Cl: 96–110 mmol/L; Ca: 2.11–2.52 mmol/L; Mg: 0.75–1.02 mmol/L; and P: 0.85–1.51 mmol/L. Total Ca rather than albumin-corrected Ca was used in the analyses, given that recent evidence suggests that unadjusted total Ca is the best and most practical alternative to ionized calcium (24). Additionally, albumin levels were included as a separate covariate in our multivariable regression models. Furthermore, patients were grouped according to the number of mineral deficiencies: those with normal mineral levels, one mineral deficiency, two mineral deficiencies, and at least three mineral deficiencies, were assigned values of 0, 1, 2, and 3, respectively.

### 2.4 Assessment of covariates

Information was collected on socio-demographic factors, disease characteristics, physical examinations conducted within 24–48 h of admission, and laboratory tests. The details are as follows:

- Socio-demographic characteristics: This includes date of birth, sex, nationality, and education level (classified as less than 12 years or 12 years and above);
- Disease characteristics: This pertains to the classification and diagnosis of diseases identified after admission and whether nutritional intervention was implemented (yes/no). The classification of diseases includes the following categories: infectious diseases, cancer, diseases of blood and hematopoietic organs, endocrine nutrition and metabolic diseases, nervous system disease, accessory organ disease, circulation system disease, respiratory disease, digestive system disease, skin and subcutaneous tissue disease, musculoskeletal disease, immune system disease, urogenital diseases, complications of pregnancy, childbirth and postpartum, chromosome abnormality, and malnutrition-related diseases;
- Physical examination: Measurements such as height and weight were taken by specialized staff in the hospitals, following standardized procedures. Weight, waist circumference, and height were measured to the nearest 0.1 kg and 0.1 cm, respectively;
- Fasting blood glucose was detected and ≥7.0 mmol/L was used to diagnose type 2 diabetes (T2D). Based on the complete blood count and renal function tests, the systemic immune-inflammation index (SII), which uses preoperative peripheral platelet count and the neutrophil-to-lymphocyte ratio (25), serum albumin, blood urea nitrogen (BUN), and serum creatinine (Scr) were considered as the covariates.

### 2.5 Statistical analysis

SAS procedures (SAS, version 9.4, SAS Institute Inc., Cary, NC, United States) were used for data analyses. All analyses were conducted at a significance level of *p* < 0.05. The normality of all continuous variables was assessed using standard probability plots and the Kolmogorov–Smirnov test. Given their non-normality, all continuous variables were presented as medians (25th and 75th percentile) and compared between groups using the Wilcoxon rank sum test. Categorical variables were described as constituent ratio or rate [frequency (%)]. The chi-square test or Fisher’s Exact Test was used for intergroup comparison of disordered variables. Preliminary analyses revealed no interactions between serum mineral levels and malnutrition by gender and age (*p* ≥ 0.6); therefore, the results are presented with both genders and age combined, adjusted for these factors.

Multivariable logistic regression models were used to explore the independent associations between serum minerals and malnutrition, as assessed by the GLIM criteria. The independent variables include K, Na, Cl, Ca, Mg, and P. In the basic models, we conducted correlational analyses to examine the relationships between serum minerals and malnutrition. Subsequently, we added potential covariates that could affect these associations. These covariates included gender, age group (18–44 years, 45–64 years, ≥65 years), years of school education (<12 years, ≥12 years), type of disease, SII, albumin, BUN, Scr, nutritional intervention (yes/no), T2D (yes/no), and other minerals (when one mineral was considered as the independent variable). Additionally, we analyzed the associations between the number of serum mineral deficiencies and malnutrition to evaluate the cumulative impact of mineral deficits.

In the subgroup analysis, we examined the relationships between serum minerals and malnutrition across age groups, genders, and education levels to assess robustness and potential variation and identify high-risk populations.

## 3 Results

### 3.1 Basic characteristics of participants

The characteristics of participants stratified by malnutrition are summarized in Table 1. A total of 3,433 adult inpatients who completed all survey documents were included in the study. The median age of all participants was 63.84 years. According to the GLIM criteria, 799 (23.27%) participants were diagnosed with malnutrition. The prevalence of malnutrition was comparable between male and female patients (24.19% vs. 22.11%, *p* = 0.2). By education level, the prevalence was similar among patients with ≥12 years and < 12 years of education (22.98% vs. 23.78%, *p* = 0.6). In contrast, the prevalence increased markedly with age: 11.90% in patients aged 18–44 years, 16.67% in those aged 45–64 years, and 32.05% in those aged ≥ 65 years (*p* < 0.0001). Those diagnosed with malnutrition tended to be older (*p* < 0.0001) and to have lower body weight (*p* < 0.0001). Additionally, they exhibited higher levels of SII and BUN (*p* < 0.0001), and lower levels of serum albumin and minerals such as K, Na, Cl, Ca, and P (all *p*-values ≤ 0.001), except for Mg, which had a *p*-value of 0.4.

**Table 1:** Characteristics^1^ stratified with different nutritional status patients by GLIM (*n* = 3,433).

Characteristic	Total sample	Malnutrition derived by GLIM^2^		
		No	Yes	*p*-value
*n* (%)	3,433	2,634 (76.73)	799 (23.27)	–
Gender (%)				
Female	1,515 (44.13)	1,180 (77.89)	335 (22.11)	0.2
Male	1,918 (55.87)	1,454 (75.81)	464 (24.19)	
Socio-demographics				
Age (years)	63.84 (53.32, 71.51)	62.00 (52.00, 69.57)	70.00 (60.00, 76.00)	<0.0001
Age (years, %)				
18–44	437 (12.73)	385 (88.10)	52 (11.90)	<0.0001
45–64	1,386 (40.37)	1,155 (83.33)	231 (16.67)	
≥65	1,610 (46.90)	1,094 (67.95)	516 (32.05)	
Educational level (years, %)				
≥12	1,253 (36.50)	955 (77.02)	298 (22.98)	0.6
<12	2,180 (63.50)	1,679 (76.22)	501 (23.78)	
Anthropometric index				
Body mass index (kg/m^2^)	24.01 (21.45, 26.47)	24.51 (22.41, 27.04)	20.87 (18.25, 24.17)	<0.0001
Weight status (%)^3^				
Normal	1,437 (41.86)	1,065 (74.11)	372 (25.89)	<0.0001
Underweight	275 (8.01)	53 (19.27)	222 (80.73)	
Overweight	1,213 (35.33)	1,049 (86.48)	164 (13.52)	
Obesity	508 (14.80)	467 (91.93)	41 (8.07)	
Biochemical Parameters				
Systemic immune-inflammation index^4^	629.60 (377.51, 1145.62)	580.36 (361.71, 989.44)	897.29 (461.41, 2028.05)	<0.0001
Serum albumin (g/L)	38.80 (37.30, 42.10)	39.70 (36.05, 42.90)	34.40 (29.70, 38.80)	<0.0001
Blood urea nitrogen (mmol/L)	5.30 (4.12, 7.01)	5.22 (4.10, 6.75)	5.71 (4.30, 8.60)	<0.0001
Serum creatinine (μmol/L)	64.00 (52.00, 79.00)	63.80 (52.50, 78.00)	64.15 (50.55, 87.45)	0.4
Serum minerals				
Potassium (mmol/L)	4.00 (3.70, 4.30)	4.00 (3.74, 4.29)	3.95 (3.58, 4.31)	0.001
Sodium (mmol/L)	139.70 (137.00, 141.90)	140.00 (137.50, 142.00)	138.00 (134.30, 141.00)	<0.0001
Chloride (mmol/L)	104.30 (101.20, 106.90)	104.80 (102.00, 107.00)	102.90 (99.00, 106.00)	<0.0001
Calcium (mmol/L)	2.25 (2.10, 2.37)	2.27 (2.14, 2.38)	2.18 (2.00, 2.32)	<0.0001
Magnesium (mmol/L)	0.90 (0.81, 1.19)	0.90 (0.81, 1.16)	0.90 (0.78, 1.28)	0.4
Phosphorus (mmol/L)	1.17 (0.97, 1.38)	1.18 (1.00, 1.38)	1.11 (0.88, 1.39)	0.0002
Nutritional intervention				
Yes	2,142 (62.39)	1,472 (68.72)	670 (31.28)	<0.0001
No	1,291 (37.61)	1,162 (90.01)	129 (9.99)	
Type 2 diabetes (%)^5^				
Yes	950 (27.67)	722 (27.41)	228 (28.54)	0.5
No	2,483 (72.33)	1912 (72.59)	571 (71.46)	

^1^Values are median (25th percentile, 75th percentile) or frequencies. Test for difference by GLIM was performed by using Wilcoxon rank-sum for non-normally distributed continuous variables and chi-square test for categorical variables.

^2^GLIM criteria for the diagnosis of malnutrition (6).

^3^Weight status was defined as Asian reference values of BMI to grade malnutrition severity according to the GLIM criteria (21, 49).

^4^SII = P × N/L, where P, N, and L were the preoperative peripheral platelet, neutrophil, and lymphocyte counts, respectively (25).

^5^Fasting blood glucose was detected and ≥7.0 mmol/L.

**Figure 1:**
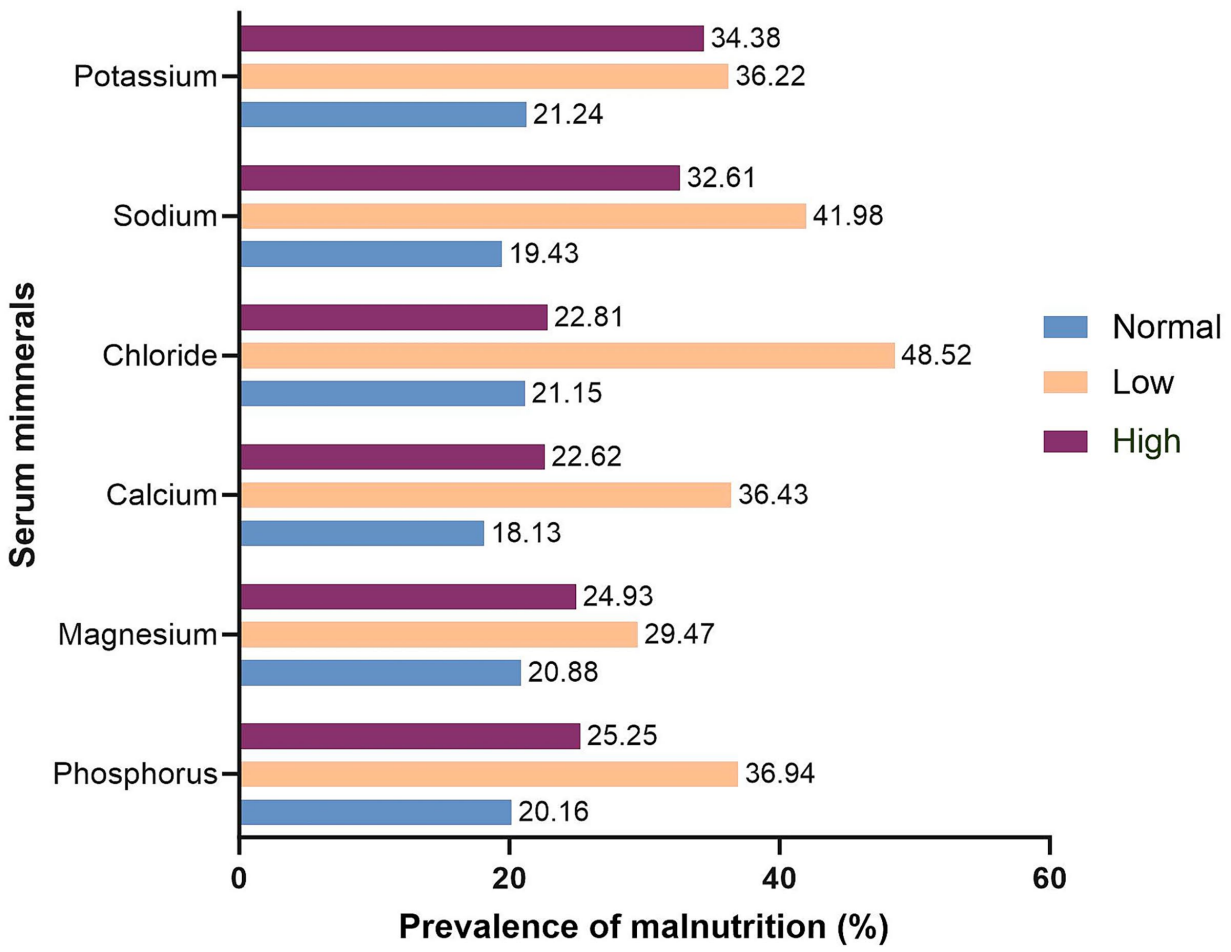
Prevalence of malnutrition across different level of serum minerals (*n* = 3,433).

We classified serum mineral levels into three categories: normal, low, and high. Our findings revealed that the prevalence of malnutrition was significantly higher among patients with hypokalemia, hyponatremia, hypochloremia, hypocalcemia, hypomagnesemia, hypophosphatemia (36.22%, 41.98%, 48.52%, 36.43%, 29.47%, and 36.94%, respectively) compared to those with normal or high levels of the corresponding minerals (*p* < 0.0001) (Figure 1). Additionally, we assessed the prevalence of mineral deficiencies and malnutrition among hospitalized patients with various disease diagnoses. Different diseases may be prone to deficiencies in other minerals, with malnutrition-related diseases being highly associated with multiple mineral deficiencies (Supplementary Figure 1). And hospitalized patients with malnutrition-related diseases, diseases of the blood and hematopoietic organs, infectious diseases, skin and subcutaneous tissue diseases, cancer, respiratory diseases, immune system diseases, and musculoskeletal diseases exhibited a particularly high-risk of malnutrition, with prevalence rates exceeding 30% across all these categories (Supplementary Figure 2).

### 3.2 Serum minerals and malnutrition

**Figure 2:**
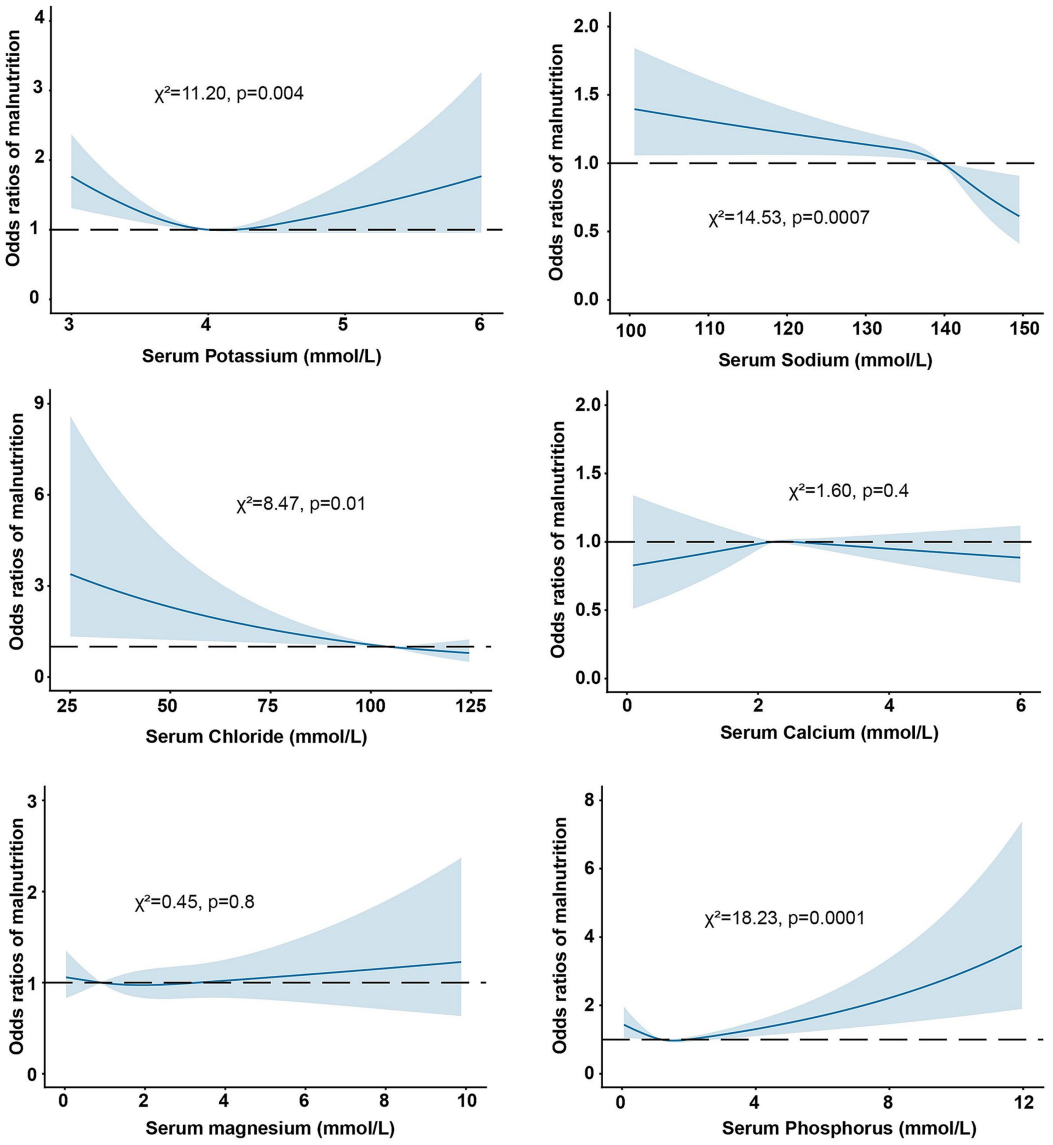
The odds ratio of malnutrition with serum minerals. OR: Odds ratio; CI: Confidence interval. Adjusted for gender, age, school year of education, type of disease, systemic immune-inflammation index, serum creatinine, blood urea nitrogen, albumin, type 2 diabetes, nutritional intervention, and other minerals (when analyzed one mineral, other minerals were adjusted for).

The relationship between six blood minerals in blood and malnutrition was examined using restricted cubic spline model analysis. K, Na, Cl, and P were significantly associated with malnutrition (*p* ≤ 0.01). Among these, K, Na, and P showed non-linear associations (K: *p* for non-linearity = 0.0007, Na: *p* for non-linearity = 0.04, P: *p* for non-linearity = 0.003) after adjustment for gender, age, school year of education, type of disease, SII, Scr, BUN, albumin, T2D, and nutritional intervention, and other minerals (when analyzing one mineral, other minerals were adjusted for). Meanwhile, no associations of Mg and Ca with malnutrition were found (Figure 2).

**Figure 3:**
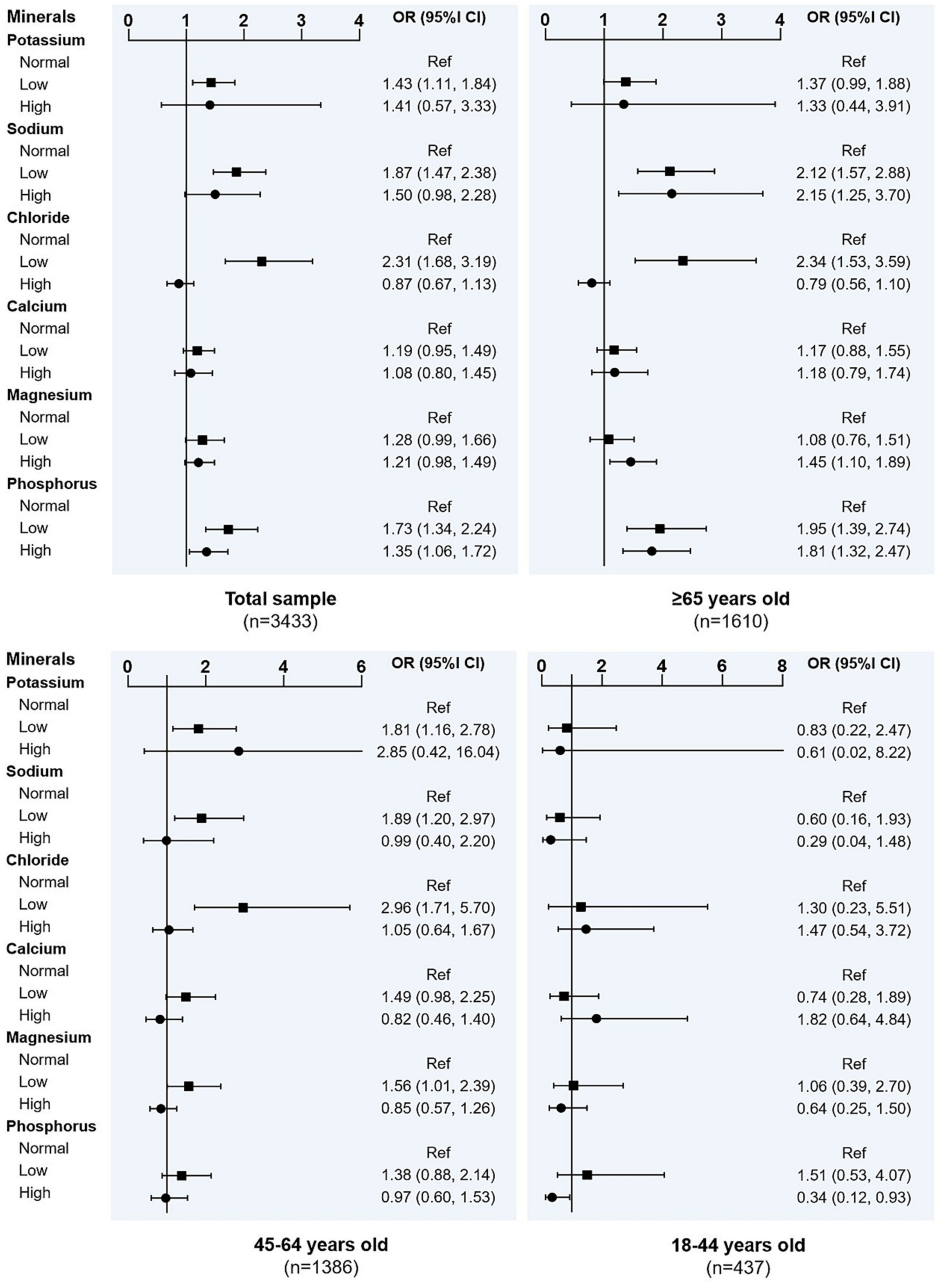
Multivariable logistic regression analysis of the association between serum minerals and GLIM-based malnutrition (*n* = 3,433). Adjusted for gender, age, school year of education, type of disease, systemic immune-inflammation index, serum creatinine, blood urea nitrogen, albumin, type 2 diabetes, nutritional intervention, and other minerals (when analyzed one mineral, other minerals were adjusted for).

We grouped the serum minerals into three categories and analyzed their associations with malnutrition, as shown in Figure 3. We found that lower levels of K, Na, Cl, and P were significantly associated with a higher likelihood of prevalent malnutrition after adjusting for potential confounding factors, with a 1.43- to 2.31-fold higher likelihood of prevalent malnutrition. Specifically, the odds ratios (ORs) and 95% CIs were as follows: K: OR (95% CI) = 1.43 (1.11, 1.84), Na: OR (95% CI) = 1.87 (1.47, 2.38), Cl: OR (95% CI) = 2.31 (1.68, 3.19), and P: OR (95% CI) = 1.73 (1.34, 2.24). Higher level of P were significantly related to a higher likelihood of prevalent malnutrition [OR (95% CI) = 1.35 (1.06, 1.72)]. No statistically significant association was observed between Ca, Mg and the likelihood of malnutrition (Ca: OR (95% CI) = 1.19 (0.95, 1.49), Mg: OR [95% CI) = 1.28 (0.99, 1.66)]. In addition, we did not observe a relationship between high serum mineral levels and prevalent malnutrition.

After stratifying inpatients by age group (18–44 years, 45–64 years, and ≥65 years), we found that significant associations were only observed among middle-aged and elderly inpatients (45–64 years and ≥65 years) after adjusting for confounding factors. Specifically, among patients aged 45–64 years, low levels of K, Na, Cl, and Mg were associated with a higher likelihood of prevalent malnutrition compared to normal levels [K: OR (95% CI) = 1.81 (1.16, 2.78), Na: OR (95% CI) = 1.89 (1.20, 2.97), Cl: OR (95% CI) = 2.96 (1.71, 5.70), Mg: OR (95% CI) = 1.56 (1.01, 2.39)]. Among inpatients aged ≥ 65 years, abnormal levels of Na and P (both low and high) were all associated with a higher likelihood of prevalent malnutrition [high Na: OR (95% CI) = 2.15 (1.25, 3.70), low Na: OR (95% CI) = 2.12 (1.57, 2.88), high P: OR (95% CI) = 1.81 (1.32, 2.47); low P: OR (95% CI) = 1.95 (1.39, 2.74)] ranging from 1.81 (1.32, 2.47) to 2.15 (1.25, 3.70). In addition, lower levels of Cl and higher levels of Mg were also associated with a higher likelihood of prevalent malnutrition among elderly patients [Cl: OR (95% CI) = 2.34 (1.53, 3.59), Mg: OR (95% CI) = 1.45 (1.10, 1.89)], whereas high Cl and low Mg showed no significant associations with malnutrition. No significant associations were observed among inpatients aged 18–44 years (Figure 3).

### 3.3 Number of low mineral levels and malnutrition

Based on the findings above, multiple low serum mineral concentrations appear to be significantly associated with malnutrition. To explore this further, the number of minerals below the normal range was calculated, and patients were categorized into four groups. Using the normal mineral level group as the reference, patients with one, two, and three or more minerals below the normal level had a respective 1.33-, 2.15-, and 2.95-fold higher likelihood of prevalent malnutrition, respectively. Specifically, for one mineral being low, the OR with a 95% CI was 1.33 (1.06, 1.66); for two minerals being low, it was 2.15 (1.65, 2.78); and for three or more minerals being low, it was 2.93 (2.14, 4.01). This indicates that increased mineral deficiencies are associated with a higher likelihood of malnutrition (Table 2).

**Table 2:** Multivariable logistic regression analysis of the association between number of hypo-minerals and malnutrition evaluated by GLIM^1^ (*n* = 3,433).

Number of hypo-minerals	Case/participants	%	Unadjusted model	Model 1^2^	Model 2^3^
Total sample					
Normal	246/1674	14.70	Ref.	Ref.	Ref.
1	223/983	22.69	1.70 (1.39, 2.08)	1.62 (1.31, 1.99)	1.33 (1.06, 1.66)
2	183/483	37.89	3.54 (2.81, 4.45)	3.14 (2.47, 4.00)	2.15 (1.65, 2.78)
3–6	147/293	50.17	5.85 (4.48, 7.63)	4.82 (3.62, 6.43)	2.93 (2.14, 4.01)
18–44 years old					
Normal	21/245	8.57	Ref.	Ref.	Ref.
1	19/126	15.08	1.89 (0.97, 3.68)	1.49 (0.71, 3.08)	1.01 (0.44, 2.26)
2	8/47	17.02	2.19 (0.86, 5.13)	2.07 (0.76, 5.22)	1.00 (0.34, 2.74)
3–6	4/19	21.05	2.85 (0.76, 8.71)	2.29 (0.57, 7.68)	1.13 (0.26, 4.11)
45–64 years old					
Normal	72/737	9.77	Ref.	Ref.	Ref.
1	55/377	14.59	1.58 (1.08, 2.29)	1.46 (0.99, 2.14)	1.27 (0.84, 1.90)
2	52/164	31.71	4.29 (2.84, 6.45)	4.13 (2.70, 6.30)	2.77 (1.75, 4.36)
3–6	52/108	48.15	8.58 (5.48, 13.47)	6.59 (4.11, 10.59)	4.11 (2.40, 7.03)
≥65 years old					
Normal	153/692	22.11	Ref.	Ref.	Ref.
1	149/480	31.04	1.59 (1.21, 2.07)	1.69 (1.29, 2.22)	1.40 (1.05, 1.87)
2	123/272	45.22	2.91 (2.16, 3.92)	2.91 (2.14, 3.98)	2.08 (1.48, 2.91)
3–6	91/166	54.82	4.27 (3.00, 6.11)	4.35 (2.97, 6.38)	2.82 (1.88, 4.26)

^1^Values are presented as odds ratio (95% confidence interval).

^2^Adjusted for gender, age (just adjusted for total sample), school year of education, type of disease, systemic immune-inflammation index, serum creatinine, blood urea nitrogen, and type 2 diabetes.

^3^Additionally adjusted for albumin and and nutritional intervention.

After stratifying by age group, the associations between low mineral levels and malnutrition were particularly pronounced among middle-aged and elderly inpatients. Specifically, among patients aged 45–64 years, those with low levels of at least three minerals had a 4.11-fold higher likelihood of prevalent malnutrition compared to those with normal levels [OR (95% CI) = 4.11 (2.40, 7.03)]. Among elderly inpatients, those with low levels of at least three minerals had a 2.82-fold higher likelihood of prevalent malnutrition [OR (95% CI) = 2.82 (1.88, 4.26)] (Table 2). We also explored the association between the number of “abnormal” serum mineral levels (either higher or lower than normal) and malnutrition. We found that the likelihood of prevalent malnutrition was slightly increased among elderly patients and slightly reduced among middle-aged patients (Supplementary Table 2).

### 3.4 Subgroup analysis

In our subgroup analysis, we examined how gender and education influence the relationship between serum minerals and malnutrition. Our findings indicate that the association between serum mineral levels and malnutrition status remained robust across sex and educational strata, irrespective of whether individual or multiple minerals were examined (Supplementary Figures 3, 4 and Supplementary Tables 3, 4).

## 4 Discussion

Malnutrition and mineral deficiencies are vital factors affecting the clinical outcomes of hospitalized patients. In our multi-center cross-sectional study, we found that serum mineral deficiencies, particularly involving K, Na, Cl, and P, are significantly associated with prevalent GLIM-defined malnutrition, with this association being most pronounced among middle-aged and elderly patients. Moreover, the cumulative burden of mineral deficiencies showed a graded association with the likelihood of prevalent malnutrition.

The diagnosis of malnutrition remains challenging, largely owing to the evolving definitions and diagnostic criteria. The GLIM criteria, established in 2019, have demonstrated superior predictive value for in-hospital complications in elderly patients compared with both the Subjective Global Assessment and ESPEN 2015 criteria (26). Additionally, these criteria serve as a sensitive and specific instrument with good concordance between screening and nutritional assessment methods (7, 27). Even when malnutrition is assessed using the GLIM criteria, considerable variation in prevalence rates remains across studies. A systematic review of 12 studies reported that the prevalence of malnutrition ranging from 16 to 80% (7). The GLIM criteria standardize the diagnosis of malnutrition through a globally unified framework that integrates phenotypic and etiologic criteria, severity staging, and etiologic classification, demonstrating robust validity and prognostic value across diverse clinical settings. Based on the GLIM criteria, our study found that the prevalence of malnutrition was 23% among hospitalized adults, higher than the 2019 level (11.6%) (28) and slightly below the national level in China (24.6%) (5).

Routine analysis of venous blood parameters can help rapidly identify malnutrition and the immediate implementation of a specialized diet (29). Numerous studies have shown that minerals are essential for maintaining cardiovascular health (13), bone health (30), COPD (14), diabetes and insulin resistance (15), adverse outcomes after ischemic stroke (31), and all-cause mortality (32). High-normal serum mineral levels are associated with a decreased risk for adverse outcomes, while low levels are linked to an increased risk. In our study, we found that up to 51% of hospitalized patients have mineral deficiencies, a higher proportion than reported in most studies, which range from 11 to 60% (9–12).

Malnutrition and mineral deficiencies are critical factors that influence clinical outcomes in hospitalized patients. Additionally, the coexistence of these issues can worsen disease development and progression, making it essential to explore their interrelationships. To date, emerging but limited research has examined the associations between mineral status and malnutrition, yet the conclusions remain inconsistent. Yilmaz et al. (11) conducted an observational study involving 156 malnourished older adults and concluded that nutritional status did not significantly correlate with multiple micronutrient deficiencies. In contrast, Yu et al. (18) analyzed blood samples from 149 patients and found that only the concentration of Ca may serve as an independent risk factor for high levels of malnutrition. Eisenhut (19) revealed that malnutrition causes a reduction in alveolar epithelial Na and Cl transport, which predisposes to death from lung injury. Yang et al. (33) reported that low Zn levels were an independent risk factor for high malnutrition risk.

In the present analysis, we found that the prevalence of malnutrition was higher among the inpatients with mineral deficiencies. Multivariable regression analysis supported these findings: deficiencies in the minerals we analyzed, particularly involving K, Na, Cl, and P, were associated with a higher likelihood of prevalent malnutrition among hospitalized adults. Moreover, our findings suggest that the cumulative burden of mineral deficiencies may be associated with the likelihood of prevalent malnutrition in a graded manner. However, this association requires further validation in prospective studies. Minerals play essential roles in a variety of basic metabolic pathways that support fundamental cellular functions. Notably, mineral deficiency can lead to malnutrition by affecting energy-yielding metabolism and disrupting bodily functions (such as immunity, digestion, and hormone regulation) (16, 34, 35), thereby preventing the body from effectively obtaining or utilizing nutrients from food. Conversely, malnutrition can exacerbate mineral deficiencies through multiple mechanisms, including reduced dietary intake, impaired intestinal absorption due to mucosal atrophy, and increased urinary losses of minerals associated with catabolic states (36). Importantly, mineral deficiencies often cluster and interact: hyperphosphatemia, hypocalcemia, and hyperkalemia are hallmark features of early hypomagnesemia (37); Mg deficiency causes refractory hypokalemia and hypocalcemia via impairing Na+/K+-ATPase function and parathyroid hormone secretion, respectively, whereas disorders of Na and Cl homeostasis disrupt Ca homeostasis through extracellular fluid volume and acid–base alterations, collectively increasing the risk of cardiac arrhythmias and metabolic bone disease in critically ill patients (38). This bidirectional and interdependent relationship creates a vicious cycle wherein mineral deficiency and malnutrition mutually reinforce one another, potentially accelerating clinical deterioration in hospitalized patients. Therefore, a balanced intake of mineral-rich foods (such as meat, legumes, vegetables, and nuts) may be an important measure to prevent malnutrition in hospitalized patients. In contrast, early nutritional intervention may simultaneously correct multiple mineral deficiency imbalances.

Additionally, we also found that high levels of Na, P and Mg were associated with a higher likelihood of prevalent malnutrition risk among elderly adults. This counterintuitive finding likely reflects that hyperphosphatemia and hypermagnesemia indicate underlying CKD and its associated protein-energy wasting (39–41), while high Na intake mirrors ultra-processed dietary patterns with low nutrient density and potential appetite suppression (42). These electrolyte abnormalities may thus serve as clinical markers of the likelihood of malnutrition rather than of nutritional adequacy (43).

In our study, malnutrition ranged from 11.9 to 32% across the age groups, with higher prevalence in older adults, likely due to age-related physiological decline, reduced access to nutritious food, and comorbidity (44). Additionally, inflammation, oxidative stress and other biochemical markers are increasingly recognized as key contributors to malnutrition in older adults (45–47). Furthermore, elderly hospitalized patients had considerably higher prevalent malnutrition levels than those among community-dwelling elderly (12.6%) (48). This suggests that implementing malnutrition screening among inpatients – with special emphasis on the geriatric population – and delivering personalized nutritional interventions is critically important. In the sub-analysis, our results revealed differences in the likelihood of malnutrition associated with serum minerals across population characteristics of the population. In particular, middle-aged and elderly inpatients (45 years and above) appeared to be more sensitive to mineral deficiencies associated with malnutrition, even after adjusting for albumin, inflammatory status, renal indices, disease type and nutritional intervention. This indicates that mineral abnormalities may capture additional nutritional vulnerability in older patients beyond what is reflected by standard inflammatory or albumin-based markers alone. Integrating routine mineral profiling into geriatric nutritional assessment protocols could therefore enhance the early identification of at-risk individuals and inform more targeted intervention strategies. Certainly, this age-stratified finding may also reflect lower event numbers and lower malnutrition prevalence in younger adults, rather than a true absence of association. Further large-scale prospective studies are warranted to examine these findings.

The present analysis had several advantages, including a large-scale study design, strong representation of the study population, standardized training for all participating nutritionists and clinicians before the study, and stringent quality control procedures used during the study. In addition, we used the GLIM criteria to assess malnutrition, which has been validated in several studies. A further strength lies in the adjustment for several confounders that potentially affected the associations between serum minerals and malnutrition, particularly socio-demographic indices, nutritional intervention mode, disease types, and inflammatory status indicators.

There are also several limitations of this study. Firstly, the cross-sectional design precludes causal inference and temporal sequencing, while the observational nature limits mechanistic insights. Secondly, although we implemented quality control measures, differences in analyzers, calibration protocols, and local laboratory standards may have introduced measurement variability. Thirdly, incomplete phenotypic data due to evolving GLIM criteria likely underestimated the prevalence malnutrition prevalence. Fourthly, despite largely comparable baseline characteristics between the included and excluded populations, persistent disparities in educational attainment suggest potential selection bias and non-random missingness. Missing mineral data may be related to hospital procedures, ward type, disease severity, and socioeconomic factors, thereby constraining the generalizability of our findings. Future studies should employ standardized laboratory protocols across all admission types to minimize missing data. Additionally, the use of total Ca in our analysis may have underestimated the true prevalence of hypocalcemia given that albumin levels were significantly lower among malnourished patients. Future studies should ideally employ direct measurements of ionized Ca for the most accurate assessment of Ca status. Although disease category was adjusted for as a covariate, substantial heterogeneity in disease types resulted in small stratum-specific sample sizes following stratification, thereby precluding disease-specific subgroup analyses of the mineral deficiency–malnutrition relationship. Furthermore, covariates such as treatment strategies, medications, intravenous fluid administration, genetic polymorphisms, dietary status, acid–base imbalance, acute illness, and comorbidity were not captured in our study, which may have influenced the association between serum minerals and malnutrition.

Our study found that serum mineral deficiencies—particularly involving potassium, sodium, chloride, and phosphorus—are significantly associated with prevalent malnutrition, with this association being most pronounced among middle-aged and elderly patients. Moreover, the cumulative burden of mineral deficiencies may be associated with the likelihood of prevalent GLIM-based malnutrition in a graded manner. However, this association requires further validation in prospective studies. Importantly, our study underscores the need to monitor mineral deficiencies in malnourished inpatients systematically and provides an evidence-based rationale for clinical evaluation and individualized management of mineral abnormalities. However, mineral supplementation should be tailored to the specific deficiency, renal function, acid–base status, concomitant medications, and overall clinical context, rather than applied uniformly.

## Data Availability

The datasets presented in this article are not readily available because the inclusion of unpublished material. Requests to access the datasets should be directed to Zengning Li, zengningli@hebmu.edu.cn.
