# Supplementary material for: Association between serum minerals and malnutrition assessed by GLIM among hospitalized adults: a multicenter cross-sectional study
Source: Front Nutr. 2026 Jul 2;13:1868158. doi: 10.3389/fnut.2026.1868158 (PMC13374522; doi:10.3389/fnut.2026.1868158)
Supplement: Supplementary file 1 [file Table_1.DOCX]

Supplementary Material

**Supplementary Table 1.** Characteristics^1^ stratified with different nutritional status patients by GLIM (n=3433).

| **Characteristics** | **Included participants** | **Excluded participants** | **p value** |
| --- | --- | --- | --- |
| **n (%)** | 3433 (66.53) | 1727 (33.47) | -- |
| **Gender** (%) |  |  |  |
| Female | 1515 (44.13) | 771 (44.64) | 0.7 |
| Male | 1918 (55.87) | 956 (55.36) |  |
| **Socio-demographics** |  |  |  |
| Age (years) | 63.84 (53.32, 71.51) | 63.92 (53.00, 71.98) | 0.6 |
| Age (years, %) |  |  |  |
| 18-44 | 437 (12.73) | 201 (11.64) | 0.5 |
| 45-64 | 1386 (40.37) | 701 (40.59) |  |
| ≥65 | 1610 (46.90) | 825 (47.77) |  |
| Educational level (years, %) |  |  |  |
| ≥12 | 1253 (36.50) | 450 (26.06) | <0.0001 |
| <12 | 2180 (63.50) | 1277 (73.94) |  |
| **Systemic Inflammation Index** | 629.60 (377.51, 1145.62) | 626.43 (377.48, 1168.99) | 0.7 |
| **Albumin** | 38.80 (37.30, 42.10) | 38.90 (34.2.0, 42.40) | 0.3 |
| **BUN** | 5.30 (4.12, 7.01) | 5.30 (4.10, 7.00) | 0.07 |
| **Cr** | 64.00 (52.00, 79.00) | 64.00 (52.20, 79.20) | 0.7 |
| **Nutritional intervention** |  |  | |
| Yes | 2142 (62.39) | 1074 (62.19) | 0.9 |
| No | 1291 (37.61) | 653 (37.81) |  |

**Supplementary Figure 1.** Prevalence of mineral deficiency among inpatients with different disease diagnosis types.

**Supplementary Figure 2.** Prevalence of malnutrition derived by GLIM among inpatients with different disease diagnosis types

**Supplementary Figure 3.** Multivariable logistic regression analysis for the association between serum minerals and malnutrition across gender (n=3433). OR: Odds ratio; CI: Confidence interval. Adjusted for gender, age, school year of education, type of disease, systemic immune-inflammation index, serum creatinine, blood urea nitrogen, albumin, type 2 diabetes, nutritional intervention, and other minerals (when analyzed one mineral, other minerals were adjusted for).

**Supplementary Figure 4.** Multivariable logistic regression analysis for the association between serum minerals and malnutrition across different education level (n=3433). OR: Odds ratio; CI: Confidence interval. Adjusted for gender, age, school year of education, type of disease, systemic immune-inflammation index, serum creatinine, blood urea nitrogen, albumin, type 2 diabetes, nutritional intervention, and other minerals (when analyzed one mineral, other minerals were adjusted for).

**Supplementary Table 2.** Multivariable logistic regression analysis for the association between number of abnormal-minerals and malnutrition evaluated by GLIM^1^ (n=3433)

|  | **Case/participants** | **%** | **Unadjusted model** | **Model 1^2^** | **Model 2^3^** |
| --- | --- | --- | --- | --- | --- |
| **Total sample** |  |  |  |  |  |
| Normal | 116/916 | 12.66 | Ref. | Ref. | Ref. |
| 1 | 153/807 | 18.96 | 1.61 (1.24, 2.10) | 1.50 (1.15, 1.97) | 1.39 (1.05, 1.84) |
| 2 | 183/743 | 24.63 | 2.25 (1.75, 2.92) | 2.08 (1.60, 2.72) | 1.92 (1.45, 2.55) |
| 3-6 | 347/967 | 35.88 | 3.86 (3.06, 4.90) | 3.26 (2.55, 4.18) | 2.40 (1.85, 3.13) |
| **18-44 years old** |  |  |  |  |  |
| Normal | 10/140 | 7.14 | Ref. | Ref. | Ref. |
| 1 | 15/113 | 13.27 | 1.99 (0.87, 4.76) | 1.38 (0.55, 3.49) | 1.44 (0.54, 3.90) |
| 2 | 13/80 | 16.25 | 2.52 (1.06, 6.20) | 2.53 (1.03, 6.42) | 1.46 (0.53, 4.05) |
| 3-6 | 14/104 | 13.46 | 2.02 (0.87, 4.88) | 1.52 (0.61, 3.85) | 0.88 (0.32, 2.40) |
| **45-64 years old** |  |  |  |  |  |
| Normal | 41/418 | 9.81 | Ref. | Ref. | Ref. |
| 1 | 41/322 | 12.73 | 1.34 (0.85, 2.13) | 1.28 (0.80, 2.05) | 1.07 (0.65, 1.76) |
| 2 | 51/296 | 17.23 | 1.91 (1.23, 3.00) | 1.80 (1.14, 2.85) | 1.49 (0.92, 2.41) |
| 3-6 | 98/350 | 28.00 | 3.58 (2.42, 5.37) | 3.03 (2.02, 4.61) | 2.07 (1.33, 3.26) |
| **≥65 years old** |  |  |  |  |  |
| Normal | 65/358 | 18.16 | Ref. | Ref. | Ref. |
| 1 | 97/372 | 26.08 | 1.59 (1.12, 2.27) | 1.63 (1.14, 2.34) | 1.59 (1.10, 2.32) |
| 2 | 119/367 | 32.43 | 2.16 (1.53, 3.07) | 2.27 (1.60, 3.25) | 2.33 (1.61, 3.40) |
| 3-6 | 235/513 | 45.81 | 3.81 (2.78, 5.28) | 3.76 (2.71, 5.28) | 3.02 (2.14, 4.31) |

^1^ Values are presented as Odds ratio (95% Confidence interval).

^2^ Adjusted for gender, age (just adjusted for total sample), school year of education, type of disease, systemic immune-inflammation index, serum creatinine, blood urea nitrogen, and type 2 diabetes.

^3^ Additionally adjusted for albumin and nutritional intervention.

**Supplementary Table 3.** Multivariable logistic regression analysis for the association between number of hypo-minerals and malnutrition across different education level and gender^1^ (n=3433)

|  | **Case/participants** | **%** | **Unadjusted model** | **Model 1^2^** | **Model 2^3^** |
| --- | --- | --- | --- | --- | --- |
| Gender |  |  |  |  |  |
| Female |  |  |  |  |  |
| Normal | 108/756 | 14.29 | Ref. | Ref. | Ref. |
| 1 | 101/437 | 23.11 | 1.80 (1.33, 2.44) | 1.60 (1.17, 2.19) | 1.29 (0.93, 1.80) |
| 2 | 64/189 | 33.86 | 3.07 (2.13, 4.41) | 2.75 (1.87, 4.03) | 1.91 (1.26, 2.86) |
| 3-6 | 62/133 | 46.62 | 5.24 (3.52, 7.80) | 4.21 (2.74, 6.47) | 2.32 (1.44, 3.71) |
| Male |  |  |  |  |  |
| Normal | 138/918 | 15.03 | Ref. | Ref. | Ref. |
| 1 | 122/546 | 22.34 | 1.63 (1.24, 2.13) | 1.63 (1.23, 2.16) | 1.43 (1.06, 1.92) |
| 2 | 119/294 | 40.48 | 3.84 (2.86, 5.17) | 3.44 (2.52, 4.71) | 2.45 (1.75, 3.43) |
| 3-6 | 85/160 | 53.13 | 6.41 (4.48, 9.20) | 5.31 (3.61, 7.85) | 3.65 (2.41, 5.54) |
| School years of education | |  |  |  |  |
| ≥12 years |  |  |  |  |  |
| Normal | 84/616 | 13.64 | Ref. | Ref. | Ref. |
| 1 | 86/357 | 24.09 | 2.01 (1.44, 2.81) | 1.92 (1.35, 2.73) | 1.42 (0.97, 2.07) |
| 2 | 70/173 | 40.46 | 4.30 (2.94, 6.31) | 3.56 (2.36, 5.38) | 2.19 (1.41, 3.40) |
| 3-6 | 58/107 | 54.21 | 7.50 (4.82, 11.74) | 5.92 (3.62, 9.72) | 3.11 (1.79, 5.44) |
| <12 years |  |  |  |  |  |
| Normal | 162/1058 | 15.31 | Ref. | Ref. | Ref. |
| 1 | 137/626 | 21.88 | 1.55 (1.20, 2.00) | 1.48 (1.14, 1.92) | 1.27 (0.96, 1.67) |
| 2 | 113/310 | 36.45 | 3.17 (2.38, 4.22) | 2.94 (2.18, 3.97) | 2.09 (1.50, 2.89) |
| 3-6 | 89/186 | 47.85 | 5.08 (3.64, 7.08) | 4.42 (3.10, 6.31) | 2.86 (1.95, 4.20) |

^1^ Values are presented as Odds ratios (95% Confidence intervals).

^2^ Adjusted for gender, age (just adjusted for total sample), school year of education, type of disease, systemic immune-inflammation index, serum creatinine, blood urea nitrogen, and type 2 diabetes.

^3^ Additionally adjusted for albumin and nutritional intervention.

**Supplementary Table 4.** Multivariable logistic regression analysis for the association between number of abnormal-minerals and malnutrition across different education level and gender^1^ (n=3433)

|  | **Case/participants** | **%** | **Unadjusted model** | **Model 1^2^** | **Model 2^3^** |
| --- | --- | --- | --- | --- | --- |
| Gender |  |  |  |  |  |
| Female |  |  |  |  |  |
| Normal | 50/398 | 12.56 | Ref. | Ref. | Ref. |
| 1 | 80/391 | 20.46 | 1.79 (1.22, 2.64) | 1.66 (1.12, 2.47) | 1.55 (1.03, 2.33) |
| 2 | 65/301 | 21.59 | 1.92 (1.28, 2.88) | 1.58 (1.04, 2.42) | 1.60 (1.04, 2.47) |
| 3-6 | 140/425 | 32.94 | 3.42 (2.40, 4.93) | 2.74 (1.89, 4.01) | 2.04 (1.38, 3.06) |
| Male |  |  |  |  |  |
| Normal | 66/518 | 12.74 | Ref. | Ref. | Ref. |
| 1 | 73/416 | 17.55 | 1.46 (1.02, 2.10) | 1.37 (0.95, 1.99) | 1.31 (0.89, 1.93) |
| 2 | 118/442 | 26.70 | 2.49 (1.79, 3.50) | 2.43 (1.74, 3.43) | 2.21 (1.54, 3.18) |
| 3-6 | 207/542 | 38.19 | 4.23 (3.12, 5.81) | 3.94 (2.88, 5.44) | 2.81 (2.00, 3.99) |
| School years of education | |  |  |  |  |
| ≥12 years |  |  |  |  |  |
| Normal | 43/369 | 11.65 | Ref. | Ref. | Ref. |
| 1 | 54/277 | 19.49 | 1.84 (1.19, 2.85) | 1.62 (1.03, 2.56) | 1.54 (0.95, 2.49) |
| 2 | 70/249 | 28.11 | 2.97 (1.95, 4.55) | 2.60 (1.68, 4.06) | 1.98 (1.23, 3.18) |
| 3-6 | 131/358 | 36.59 | 4.38 (3.00, 6.48) | 3.44 (2.30, 5.22) | 2.37 (1.53, 3.69) |
| <12 years |  |  |  |  |  |
| Normal | 73/547 | 13.35 | Ref. | Ref. | Ref. |
| 1 | 99/530 | 18.68 | 1.49 (1.08, 2.08) | 1.44 (1.03, 2.02) | 1.33 (0.93, 1.89) |
| 2 | 113/494 | 22.87 | 1.93 (1.40, 2.67) | 1.81 (1.30, 2.53) | 1.81 (1.28, 2.57) |
| 3-6 | 216/609 | 35.47 | 3.57 (2.66, 4.83) | 3.16 (2.33, 4.34) | 2.40 (1.73, 3.35) |

^1^ Values are presented as Odds ratios (95% Confidence intervals).

^2^ Adjusted for gender, age (just adjusted for total sample), school year of education, type of disease, systemic immune-inflammation index, serum creatinine, blood urea nitrogen, and type 2 diabetes.

^3^ Additionally adjusted for albumin and nutritional intervention.
